# Preparation and Characterization of Ginger Peel Polysaccharide–Zn (II) Complexes and Evaluation of Anti-Inflammatory Activity

**DOI:** 10.3390/antiox11122331

**Published:** 2022-11-25

**Authors:** Wenwen Li, Zhichang Qiu, Yue Ma, Bin Zhang, Lingyu Li, Qiulin Li, Qiuxia He, Zhenjia Zheng

**Affiliations:** 1 Key Laboratory of Food Processing Technology and Quality Control of Shandong Higher Education Institutes, College of Food Science and Engineering, Shandong Agricultural University, Tai’an 271018, China; 2 Beijing Academy of Agriculture and Forestry Sciences, Beijing Key Laboratory of Agricultural Products of Fruits and Vegetables Preservation and Processing, Key Laboratory of Vegetable Postharvest Processing, Institute of Agri-Food Processing and Nutrition, Ministry of Agriculture and Rural Affairs, Beijing 100097, China; 3 School of Materials Science and Engineering, Suzhou University of Science and Technology, Suzhou 215011, China; 4 Science and Technology Service Platform of Shandong Academy of Sciences, Qilu University of Technology (Shandong Academy of Sciences), Jinan 250103, China

**Keywords:** ginger peel polysaccharide–Zn (II) complexes, synthesis, structural characterization, anti-inflammatory activity, zebrafish

## Abstract

The present study aimed to explore the improvement of the bioactivity of ginger peel polysaccharides (GPs) by the modification of zinc after structural characterization. The obtained GP–Zn (II) complexes consisted dominantly of glucose and galactose in a mass proportion of 95.10:2.10, with a molecular weight of 4.90 × 10^5^ Da and a Zn content of 21.17 mg/g. The chelation of GPs and Zn (II) was mainly involved in the O–H of hydroxyl groups, and this interaction reduced the crystallinity and decreased the asymmetry of GPs, with a slight effect on the thermal stability. The administration of GPs and their Zn (II) complexes effectively alleviated CuSO_4_-induced inflammatory response in zebrafish (*Tg*: *zlyz-EGFP*) *via* down-regulating the mRNA expression levels of pro-inflammatory cytokines (IL-1*β*, IL-6, IL-8, IL-12 and TNF-*α*) and upregulating the expression of anti-inflammatory cytokine (IL-10). Furthermore, the modification of Zn (II) enhanced the inflammation-inhibiting effect of polysaccharides. Therefore, GP–Zn (II) complexes could be applied as a candidate anti-inflammatory agent for the treatment of chronic inflammation-related diseases.

## 1. Introduction

Plant polysaccharides have drawn great public interest in the field of food and pharmaceuticals because of their diverse health benefits (e.g., anti-inflammatory activity, antioxidant activity, intestinal flora-regulating activity) [1,2,3]. In particular, the inflammation-inhibiting effect of plant polysaccharides has become the research focus. Accumulating studies have demonstrated that *Rehmannia glutinosa* polysaccharides [4], *Ginkgo biloba* polysaccharides [5] and *Phellinus baumii* polysaccharides [6] possess excellent anti-inflammatory activity.

The anti-inflammatory effect of polysaccharides depends on their structural features [7]. Polysaccharides readily react with other compounds because they have a large surface area and reactive sites containing hydroxyl, carboxyl and other groups. These exposed sites also provide a basis for chemical modifications [8]. The chemical modification of polysaccharides by changing the structure or adding new groups can boost the existing bioactivities or bring new functional properties [9,10]. Therefore, it is a potential method to enhance the anti-inflammatory activity of polysaccharides by chemical modifications. Zinc (Zn [II]) is considered to be an indispensable microelement that is involved in the synthesis and metabolism of many enzymes (such as superoxide dismutase and alkaline phosphatase) [11]. Furthermore, it also exhibits excellent anti-inflammatory properties. Accordingly, the approximate Zn (II) intake can reduce oxidative stress and inflammatory cytokine production and improve the immunity of the body [12]. Zn (II)-containing polysaccharides are Zn (II) supplements that are less naturally occurring but are being enthusiastically developed by researchers [13,14]. Zhao et al. (2017) prepared a *Flammulina velutipes* polysaccharide–Zn complex, which exhibited a better inhibitory effect than *F*. *velutipes* polysaccharides on the lipopolysaccharide (LPS)-induced secretion of inflammatory markers (TNF-*α*, INF-*γ*, IL-6 and NO) in RAW 264.7 cells [15]. We speculated that the modification of Zn (II) could be a promising way to improve the biological activity of the original polysaccharides due to the dual or synergistic effects. Ginger (*Zingiber officinale* Roscoe) belongs to the genus *Zingiber* in the family of *Zingiberaceae* and is consumed as a vegetable and a condiment. During the processing of ginger, a large amount of peel waste is produced, which can waste resources and pollute the environment once discarded [16]. Polysaccharides are the predominant nutritional components of ginger peel and have high pharmacological effects [17]. Although polysaccharides from ginger have been extensively reported, there is a lack of attention on the structure, bioactivity and Zn modification of polysaccharides from ginger peel.

In this study, ginger peel polysaccharide (GP–Zn (II) complexes were synthesized through thermal reaction and characterized by high-performance anion-exchange chromatography coupled with pulsed amperometric detector (HPAEC-PAD), high-performance gel permeation chromatography equipped with refractive index detector (HPGPC-RID), scanning electron microscopy (SEM), ultraviolet-visible (UV-vis) spectroscopy, Fourier transform infrared-attenuated total reflection (FTIR-ATR) spectrometry, X-ray diffraction (XRD), circular dichroism (CD) spectroscopy and thermal analysis. Moreover, the anti-inflammatory effects of GPs and their Zn (II) derivatives were evaluated based on a CuSO_4_-induced inflammation model in zebrafish, and the possible underlying mechanisms were explored by quantitative real-time polymerase chain reaction (qRT-PCR). The findings are expected to facilitate the application of GP–Zn (II) complexes in the food and pharmacological industries as an inflammatory agent.

## 2. Materials and Methods

### 2.1. Material and Reagents

Ginger (*Zingiber officinale* Roscoe) peel powder was obtained from Qujing city (Yunnan, China). The monosaccharide standards, pullulan standards (342–805,000 Da) and dialysis bags (500 Da and 2000 Da) were purchased from Shanghai Yuanye Bio-Technology Co., Ltd. (Shanghai, China). Zn (II) standard solution was obtained from Guobiao Testing & Certification Co., Ltd. (Beijing, China). ZnSO_4_·7H_2_O, CuSO_4_, sodium acetate (NaAC), acetonitrile and trifluoroacetic acid (TFA) were provided by Sigma-Aldrich (St. Louis, MO, USA). All other reagents and chemicals were of analytical grade.

### 2.2. Extraction and Purification of Polysaccharides from Ginger Peel

As reported by Zhou, Huang, and Chen (2021), polysaccharides were isolated and purified from ginger peel by hot-water extraction method [18]. Briefly, the ginger peel powder was dissolved in a conical flask in a ratio of 1:10 (*w*/*v*) and then incubated twice in a water bath at 80 °C for 2 h. The resulting mixture was centrifuged (6000× *g*, 10 min) using a TGL 16 centrifuge (Changsha Yingtai Instrument Co., Ltd., Changsha, China) and concentrated using an N-1000 EYELA rotary evaporator (Tokyo Rikakikai Co., Ltd., Tokyo, Japan) at 60 °C. After polysaccharide-containing solutions were deproteinized using Sevag reagent (chloroform: butyl alcohol = 4:1, *v*/*v*), the purified polysaccharides were collected by precipitation using 4-fold volume of absolute ethanol, dialyzed by dialysis (500 Da) and then freeze-dried using a SCIENTZ-12N freeze-dryer (Ningbo SCIENTZ Biotechnology Co., Ltd., Ningbo, China) for the preparation of GP–Zn (II) complexes. A total of 25.58 g purified GPs was obtained through the above extraction and purification procedures, with a yield of 11.79%.

### 2.3. Synthesis of GP–Zn (II) Derivatives

According to the previous study in our laboratory, polysaccharide solution and ZnSO_4_ solution were used to prepare GP–Zn (II) complexes under heating conditions [19]. GPs and ZnSO_4_·7H_2_O were first dissolved into aqueous solutions of 24 mg/mL and 1 mg/mL, respectively. Then, the ZnSO_4_·solution was added dropwise to the polysaccharide solution at 50 °C under continuous stirring, with pH maintaining at about 8 using 1.0 mol/L NaOH solution. After reaction at 50 °C for 125 min, they were cooled (4 °C, 10 min), centrifuged (4000× *g*, 10 min) and precipitated (4 °C, 12 h) with absolute ethanol in a ratio of 4:1 (*v*/*v*). Finally, the collected precipitates were reconstituted in deionized water, followed by dialysis, concentration and freeze-drying to obtain GP–Zn (II) complexes. Total sugar content and protein content were measured by the phenol-sulfuric acid method and the Bradford method, and Zn (II) content was determined using an Agilent 7800 inductively coupled plasma mass spectrometer (Agilent Technologies, Santa Clara County, CA, USA) [20].

### 2.4. Monosaccharide Composition

As reported by Li et al. (2021), the monosaccharide composition of GPs and their Zn (II) derivatives was determined using an ICS-5000^+^ HPAEC-PAD system (Thermo Fisher Scientific, Waltham, MA, USA) [21]. Briefly, the acid hydrolysis of GPs and their Zn (II) derivatives was carried out by incubating 2 mL of TFA (2.0 mol/L) and 10 mg of sample at 80 °C for 3 h. Then, the hydrolysate was rotary evaporated to remove residual TFA. Prior to injection into a Dionex™ AminoPac™ PA10 IC column (3 × 250 mm) and analysis using ultrapure water, NaOH solution (0.20 mol/L) and NaAC solution (1.0 mol/L), the mixture was purified using Supelclean™ ENVI-18 tubes and 0.22 μm millipore filters.

### 2.5. Molecular Weight Distribution

Two lyophilized polysaccharide samples were first redissolved in deionized water to a concentration of 5 mg/mL and purified using 0.22 μm millipore filters. Then, the molecular weight distribution was analyzed using an LC-20A system (Shimadzu, Kyoto, Japan) equipped with tandem chromatographic columns (Shodex OHpak SB-802HQ, SB-802.5HQ, SB-803HQ gel columns; 300 × 38 mm, 6 μm) [21]. The elution was conducted using 0.3 mol/L NaNO_3_ at constant temperature (30 °C) and flow rate (0.3 mL/min). Pullulan standards with different molecular weights were used to draw the calibration curve.

### 2.6. SEM Analysis

The surface morphology of GPs and their Zn (II) derivatives was examined using a Zeiss-SUPRATM 55 SEM (Oberkochen, Germany) [22]. The lyophilized polysaccharide particles were fixed on the SEM specimen stub and sputter-coated with gold. Images were obtained at an acceleration voltage of 5 kV and at magnifications of 200× and 1000×, respectively.

### 2.7. UV-Vis and FTIR Spectral Analyses

GPs and their Zn (II) derivatives were redissolved using deionized water to a final concentration of 2 mg/mL and then scanned in the UV full wavelength range of 190–600 nm using a UV-2450 spectrophotometer (Shimadzu, Japan), respectively. Three scans were performed for each sample.

The structural features of GPs and GP–Zn (II) complexes were elucidated using a Thermo Nicolet IS10 FTIR spectrometer equipped with a universal ATR accessory (Thermo Fisher Scientific Inc., Waltham, MA, USA). As reported by Li et al. (2021), 10.0 mg of dried polysaccharide samples were directly compressed on the accessory manipulator, followed by scanning from 4000 cm^−1^ to 400 cm^−1^ at a resolution of 4 cm^−1^, in triplicate [21]. The background spectrum of air was subtracted before the absorption bands in the FTIR spectra were annotated using OMNIC 8.2 software.

### 2.8. XRD Analysis

The EMPYREAN X-ray diffractometer (Panalytical B.V., Almelo, The Netherlands) was used to determine the crystal properties of the original and modified GPs at 25 °C. The lyophilized GPs and GP–Zn (II) complexes were fully ground and sieved into fine powder (40 μm). They were then spread evenly on circular transmission holders between X-ray transparent polymer foils. Data collection was performed using a Cu Kα radiation source from a diffraction angle (2θ) of 5° to 80° at a scan rate of 4°/min [22].

### 2.9. CD Spectral Analysis

The secondary structures of GPs and their Zn (II) derivatives were analyzed using a Chirascan plus ACD spectropolarimeter (Applied Photophysics, Leatherhead, UK). Two polysaccharides were dissolved in 5 mM citrate buffer solution and then scanned in the range of 180–400 nm at a constant rate (100 nm/min) and bandwidth (1.0 nm). Three scans were conducted and averaged for each sample.

### 2.10. Thermal Analysis

The simultaneous thermal analyzer (Netzsch STA 449F3 Jupiter, Selb, Germany) was used to examine the thermodynamic properties of GPs and their Zn (II) derivatives, including thermogravimetry (TG) and derivative thermogravimetry (DTG) [22]. Two dried polysaccharide powders were hermetically sealed into standard aluminum pans and then heated to 600 °C at a rate of 10 °C/min under the protection of nitrogen (20 mL/min).

### 2.11. Anti-Inflammatory Effects of GPs and Their Zn (II) Derivatives Based on CuSO_4_-Induced Zebrafish Model

#### 2.11.1. Effect of GPs and Their Zn (II) Derivatives on the Migration of Macrophages

The transgenic zebrafish (*Tg: zlyz-EGFP*) with enhanced green fluorescently labeled inflammatory cells were obtained from the Shandong Academy of Sciences (Jinan, China). They were kept in a standard feeding environment (28 °C, 14:10-h light-dark cycle), with *Artemia salina* provided as the regular diet. As reported by Zhu et al. (2022), the embryos were produced by co-culturing equal numbers of sexually mature male and female zebrafish for 12 h [22]. After the collected embryos were sterilized with 0.1% methylene blue, they were checked under an AXIO Zoom.V16 stereomicroscope (Carl Zeiss, Jena, Germany). The naturally developing embryos at 72 h post-fertilization (hpf) were selected and placed into individual wells of 6-well plates for subsequent experiments.

All zebrafish embryos were randomly assigned to nine groups (*n* = 30/well): the blank group, model group, positive control group (supplemented with 20 μmol/L ibuprofen in embryo culture medium) and six polysaccharide or its Zn (II) complex-treated groups (supplemented with GPs and GP–Zn (II) derivatives at low [5 µg/mL], medium [10 µg/mL] and high [100 µg/mL] doses in embryo culture medium, respectively), in triplicate. After all treatment groups were incubated at 28 °C under light for 3 h, 40 μM CuSO_4_ was applied to zebrafish embryos (except the control group) for 1 h under light-proof conditions. Finally, the inflammatory responses were observed under an SZX16 fluorescence microscope (Olympus, Tokyo, Japan), and the inflammatory cells that migrated and accumulated to the lateral line were quantified using Image-Pro Plus software (version 6.0) [22].

#### 2.11.2. Effect of GPs and Their Zn (II) Derivatives on the Expression Levels of Inflammation-Associated Cytokines

The zebrafish embryos were rinsed with fresh water, before the extraction of their total RNA using a commercial total RNA extraction kit (Vazyme, RC101-01). After the reverse transcription was performed for the synthesis of complementary DNA (cDNA) using extracted RNA and commercial premixes (Vazyme, R323-01), qRT-PCR was performed using a Roche LightCycler 96 RT-PCR system.

Six primers were obtained from Shanghai BioSune Biotechnology Co., Ltd. (Shanghai, China), and their detailed sequences are shown in Table S1. The reaction system consisted of 10 μL of 10 × ChamQ Universal SYBR qPCR Master Mix, 1 μL of cDNA, 0.4 μL of forward and reverse primers respectively, (10 μM) and 8.2 μL of ddH_2_O (with a total volume of 20 μL). The qRT-PCR reaction pattern was as follows: pre-denaturation at 95 °C for 30 s, 40 cycles of 95 °C for 10 s and 60 °C for 30 s, followed by 1 cycle of 95 °C for 15 s, 60 °C for 60 s and 95 °C for 15 s. According to the fluorescent signal intensity, the expression levels of six inflammatory cytokines were quantified by the comparative 2^−ΔΔCt^ method, and *β*-actin was used as the internal control.

### 2.12. Statistical Analysis

All experimental results were expressed as mean ± standard deviation. One-way analysis of variance (ANOVA) combined with Tukey’s test was used to assess the significance of differences by IBM SPSS Statistics software (version 28) at *p* < 0.05.

## 3. Results and Discussion

### 3.1. Preparation of GP–Zn (II) Complexes

GP–Zn (II) complexes were synthesized according to our previously optimized technological conditions [19]: GPs to Zn^2+^ ratio of 24:1 (*w*/*w*), temperature of 60 °C, pH of 8.0 and time of 125.0 min, with the highest chelation rate of 98.53 ± 0.31%. The total sugar content and total protein content of GPs were 94.27% and 1.76%, which were changed to 96.04% and 0.16% after chelation with Zn (II). The inductively coupled plasma mass spectrometry indicated that the Zn (II) content of the GP–Zn (II) complexes was 21.17 ± 0.25 mg/g.

### 3.2. Monosaccharide Composition

**Figure 1:**
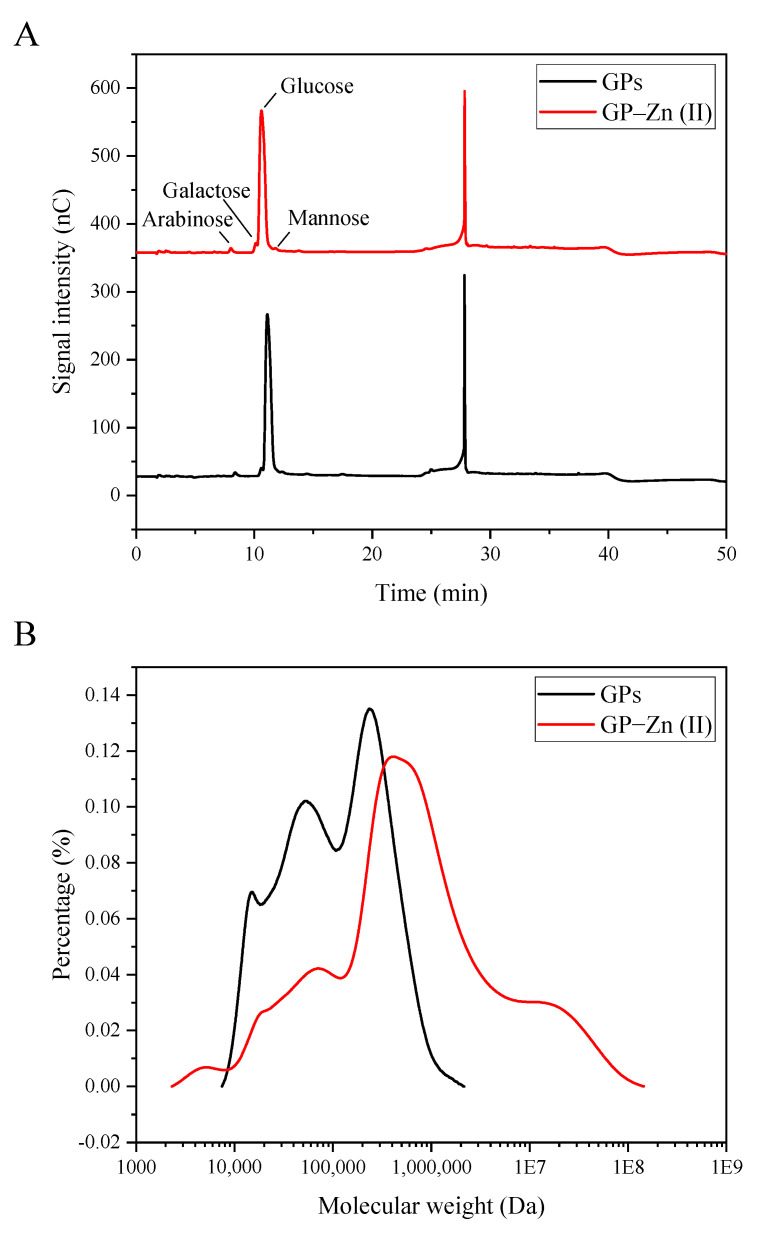
Monosaccharide profile (**A**) and molecular weight distribution (**B**) of ginger peel polysaccharide (GPs) and their Zn (II) derivatives.

Figure 1A showed that the GPs were composed of glucose, galactose, mannose and arabinose, with a mass ratio of 96.20:1.50:1.30:1.00. After the chelation of Zn (II), the monosaccharide types remained unchanged, but the relative proportion of GP–Zn (II) complexes changed to 95.10:2.10:1.60:1.20. Compared with GPs, the mass ratio of glucose in GP–Zn (II) complexes was significantly reduced, indicating that the binding sites of Zn (II) might occur mainly on glucose residues [23]. Jia et al. (2021) found that there were different decreases in the glucose content in corn silk polysaccharide (CSP)–Fe (III), CSP–Zn (II) and CSP–Cr (III) complexes compared with CSPs [23]. In *Fritillaria ussuriensis* polysaccharide (FUP)–Zn derivatives with different degrees of substitution, lower levels of glucose were also observed in the monosaccharide profile [14]. The ginger polysaccharides are dominated by neutral polysaccharides. As reported by Yang et al. (2021), ginger neutral polysaccharides belonged to a *α*-glucan containing the backbone of 1,4-linked *α*-D-Glc*p* and the branched chain of α-D-Glc*p* residues at the C-6 position [24]. The abundant hydroxyl groups on the GP chains provided sufficient reactive sites for the chelation of GPs and Zn (II). Therefore, the binding of hydroxyl groups on glucose to Zn (II) led to a decrease in its relative level.

### 3.3. Molecular Weight Distribution

Figure 1B indicated that both GPs and their Zn (II) derivatives exhibited a relatively wide molecular weight distribution. This was due to the absence of further fractionation of the purified polysaccharides. According to the calibration curve, the weight-average molecular weight of GPs was determined to be 1.50 × 10^5^ Da, which was significantly increased to 4.90 × 10^5^ Da after the modification by Zn (II). This suggested that the metal ions promoted the connection of polysaccharide molecules [23]. Furthermore, the chelation of Zn (II) resulted in a significant increase in the polydispersity index (Mw/Mn) of GPs from 3.55 to 6.23. From the molecular weight distribution curves of GPs and their Zn (II) complexes, the increase in polydispersity index was mainly due to the appearance of high molecular weight polymers. Therefore, the relatively wider Mw distribution was caused by simultaneous conjugation of one Zn (II) with more than one GP molecule.

### 3.4. Morphological Characteristics

**Figure 2:**
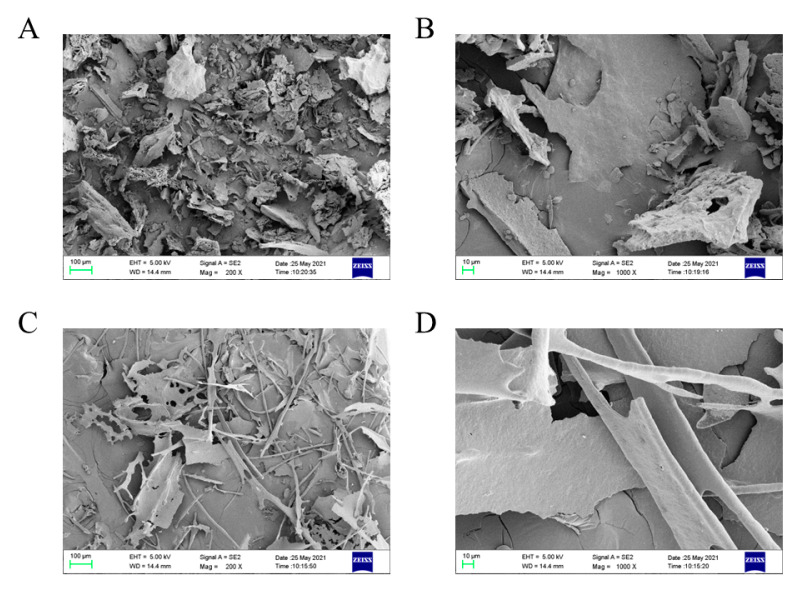
Morphological features of GPs and their Zn (II) derivatives. (**A**,**B**) SEM images of GPs at magnifications of 200× and 1000×, respectively; (**C**,**D**) SEM images of GP–Zn (II) complexes at magnifications of 200× and 1000×, respectively.

The surface morphological features of GPs and their Zn (II) derivatives were evaluated by SEM. As shown in Figure 2, GPs appeared as irregular fragments with rough and porous surface under 200× magnification. After the modification of Zn (II), the morphological characteristics of polysaccharide derivatives were greatly different from those of GPs, exhibiting a sheet-like structure and some dendritic fragments. Under 1000 × magnification, the surface of GPs was rough and rugged, while GP–Zn (II) complexes exhibited a compact appearance with a relatively flat surface. The morphological features of polysaccharides are closely correlated with the chemical structure (e.g., glycosidic linkage and chain conformation). Therefore, the changes in the surface morphology of GPs after Zn (II) chelation were mainly attributed to the interaction of polysaccharides with metal ions, which confirmed the successful attachment of Zn (II) to polysaccharide chains and the formation of their complexes [14,23].

### 3.5. UV-Vis and FTIR Spectral Analyses

**Figure 3:**
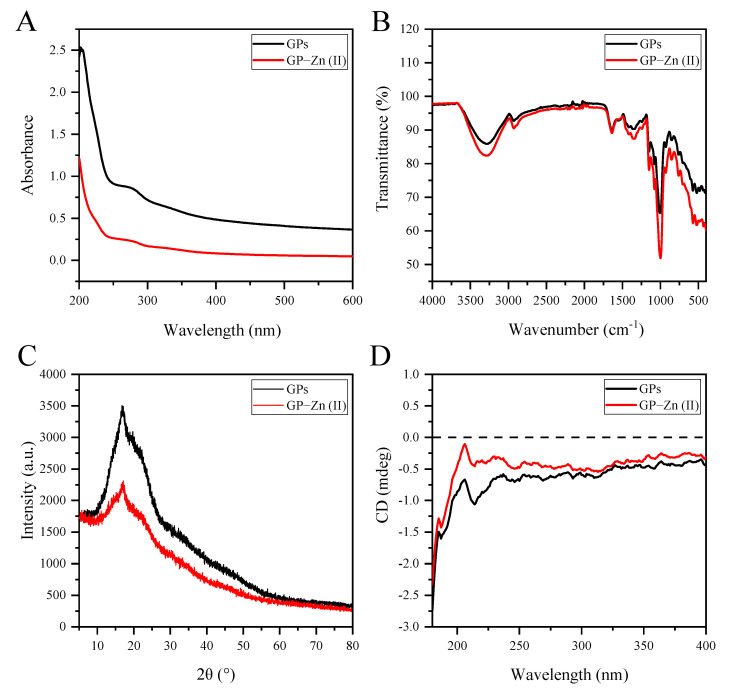
UV-vis spectra (**A**), FTIR spectra (**B**), XRD patterns (**C**) and CD spectra (**D**) of GPs and their Zn (II) derivatives.

The UV-vis spectra of GPs and their Zn (II) derivatives were shown in Figure 3A. The absence of absorption peaks at 260 nm and 280 nm suggested that there were very low contents of proteins and nucleic acids in GPs and their Zn (II) derivatives. This was in agreement with the data of chemical properties. Furthermore, the UV absorbance values of GP–Zn (II) complexes were considerably lower compared with GPs, which was also observed in the UV-vis spectra of CSP–Zn (II) complexes, CSP–Cr (III) complexes and FUP–Zn (II) complexes [14,23]. Some researchers have attributed this phenomenon to the reduction of the conjugated system caused by the chelation of the polysaccharides and metal ions (e.g., Zn [II] and Cr [III]) through hydroxyl bonding [14,23]. As mentioned earlier, neutral polysaccharides were the main polysaccharides in ginger, accompanied by small amounts of acidic polysaccharides (pectins) [25]. Therefore, the binary or quaternary conjugated system formed by Zn (II) and carboxyl groups from these acidic polysaccharides might be partially responsible for the reduced UV absorption intensity. However, there were some other factors that needed to be further investigated.

FTIR spectra provided information about the functional groups in the polysaccharide structures. As shown in Figure 3B, GP–Zn (II) complexes had a similar absorption pattern with GPs in the FTIR spectra, indicating that the chelation of GPs and Zn (II) did not alter the basic structure or functional group distribution of the polysaccharide chains. The strong absorption signal in the range of 3600–3200 cm^−1^ was caused by O–H stretching vibrations, while the weak absorption signal at 2900–3000 cm^−1^ was mainly ascribed to asymmetric C–H stretching vibrations [25]. The C–O–C and O–C–O stretching vibrations of glycosidic linkages and rings or C–O–H stretching vibrations of side groups in polysaccharides led to absorption bands from 1000.0 cm^−1^ to 1200.0 cm^−1^ [22]. These absorption signals were typical features of polysaccharides [26]. After chemical modification, the characteristic bands of the hydroxyl groups had a shift from 3292.1 cm^−1^ to 3274.2 cm^−1^ and an increase in the absorption intensity, suggesting the formation of new hydrogen bonds. This change was also found in the FTIR spectra of FUP–Zn complexes [14]. The absorption bands at 995.8/1003.5 cm^−1^, 1078.2/1077.5 cm^−1^ and 1147.8/1148.8 cm^−1^ indicated the presence of pyranose rings [25,26], and the bands at 852.1/853.0 cm^−1^ and 928.2/929.6 cm^−1^ suggested that *α*- and *β*-glycosidic bonds were co-existing in polysaccharides and their Zn (II) complexes [24,27]. Yang et al. (2021) reported that the neutral polysaccharides from ginger contained the residues of (1,4)-*α*-D-Glc*p*, (1,4,6)-*α*-D-Glc*p*, terminal *α*-D-Glc*p* and *β*-D-Glc*p* based on methylation analysis and 1D/2D nuclear magnetic resonance spectroscopy [24]. This confirmed our analysis for the FTIR spectra. Furthermore, the intensity of the typical absorption signal of Zn (II) at 424 cm^−1^ was greatly increased in the GP–Zn (II) complexes compared with GPs, which might be derived from the stretching vibration of Zn–O. These results confirmed that GPs interacted with Zn (II) *via* hydrogen bonds.

### 3.6. XRD Analysis

The crystalline or amorphous nature of the polysaccharides was examined by XRD analysis, according to the shape of the diffraction peaks [28]. Figure 3C showed that GPs had a broad diffraction peak at 2θ of 20°, indicating that they were amorphous materials in nature. After chemical modification by Zn (II), the crystalline properties of GPs remained unchanged, but the intensity of the diffraction peaks was significantly decreased. This might be attributed to the interaction of Zn (II) and the functional groups of GPs, which affected the hydrogen bonding interactions (essential for the formation of crystals) and led to the stereochemical blocking of the polysaccharide chains [22,23]. Therefore, the chelation of GPs and Zn (II) caused a slight decrease in the crystalline of GPs, which might affect the solubility, flexibility and swelling capacity of GP–Zn (II) derivatives [13].

### 3.7. CD Spectral Analysis

CD spectroscopy provides a rapid and accurate characterization of the conformational changes in biological macromolecules due to different contributions to the static polarizability and the orientation of the chromophores [23,29]. Figure 3D illustrated that negative Cotton effects were observed at about 190 nm and 210 nm in the CD spectra of GPs and their Zn (II) derivatives, indicating that they both existed in aqueous solution as irregular curls [23]. Although each positive and negative peak appeared at similar absorption positions, the modification of Zn (II) into the polysaccharide structure caused some changes in the peak intensities of CD spectra. For examples, GP–Zn (II) complexes had a lower negative Cotton effect (the difference between the molar absorption coefficients of left and right circularly polarized light) at around 190 nm and 210 nm. The reduced asymmetry indicated that the formation of Zn–O bonding during the chemical modification altered the conformation of GPs [30].

### 3.8. Thermal Analysis

**Figure 4:**
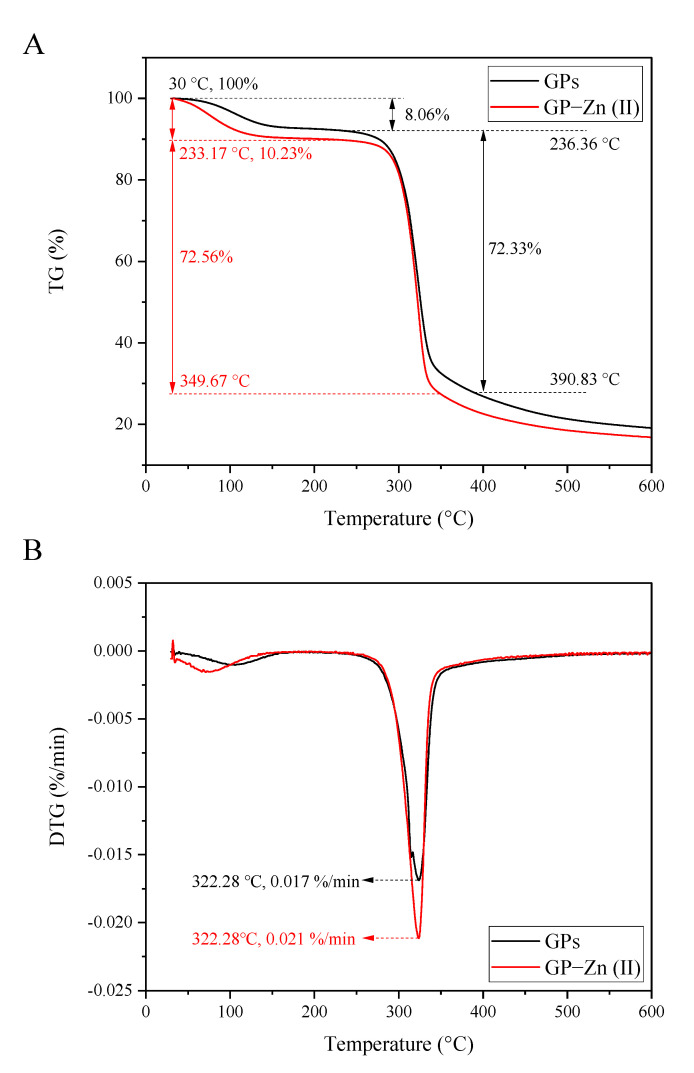
Thermodynamic analysis of GPs and GP–Zn (II) complexes. (**A**) TG curves; (**B**) DTG curves.

Thermodynamic analysis is essential for drug development and application [31]. As presented in Figure 4A,B, two biomacromolecules showed similar thermodynamic behaviors in the range of 30–600 °C. In terms of TG curves, the first phase of the thermal decomposition process was related to the loss of bound water, where GP–Zn (II) derivatives had a slightly higher mass loss rate (10.23%) than that of GPs (8.06%) in the range of 30–241 °C. The decarboxylation and thermal decomposition of GPs and their Zn (II) derivatives triggered the second stage of mass loss (>250 °C), resulting in 72.33% and 72.56% of mass loss, respectively. In DTG curves, the first endothermic peak of GPs had a slightly higher temperature, compared with their Zn (II) derivatives. However, the maximum decomposition rate occurred at the same temperature (322.28 °C). These results indicated that the thermal stability of GPs was slightly affected by the modification of Zn (II).

### 3.9. Evaluation of the Anti-Inflammatory Activity of GPs and Their Zn (II) Derivatives and Underlying Mechanisms

#### 3.9.1. Anti-Inflammatory Effects Based on CuSO_4_-Induced Zebrafish Model

In this study, the anti-inflammatory activity of GPs and their Zn (II) complexes was evaluated by visualizing the chemotactic migration of leukocytes in response to acute inflammation using transgenic zebrafish (*Tg*: *zlyz-EGFP*) as an *in vivo* model. In transgenic zebrafish (*Tg*: *zlyz-EGFP*) embryos, EGFP-positive cells mainly contain subsets of monocytes/macrophages and neutrophils, both of which are known as ‘leukocytes’ and play an important role in acute inflammation [32]. For the control group, leukocytes in zebrafish are mainly distributed in the dorsal aorta and posterior cardinal veins in the trunk region, and little macrophage migration is observed. Once exposed to sublethal concentrations of CuSO_4_, these inflammatory cells can rapidly migrate and accumulate to the lateral line, which positively correlates with the magnitude of the inflammatory response [22,32]. In comparison, the compounds that can modulate the migratory behavior of leukocytes are considered as anti-inflammatory drugs [22,32]. Therefore, the inflammatory cells (EGFP-positive cells) that migrate and accumulate to the lateral line neuromasts are observed and quantified to determine the inhibition-inhibiting activity of GPs and GP–Zn (II) complexes.

**Figure 5:**
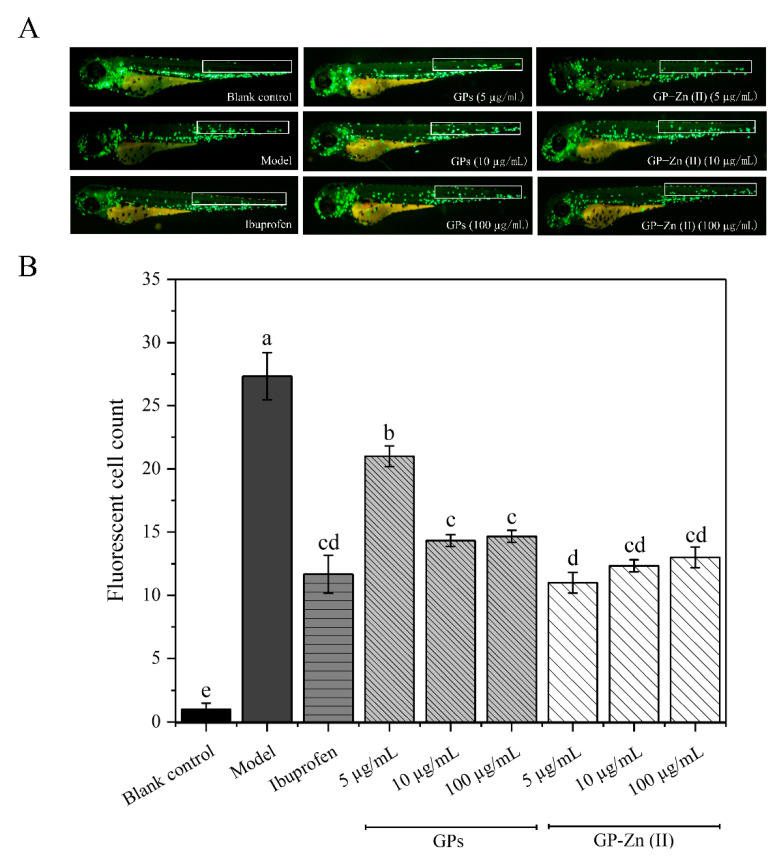
Alleviation of CuSO_4_-induced inflammatory response in zebrafish by GPs and their Zn (II) derivatives. (**A**) Representative images of zebrafish treated with CuSO_4_, ibuprofen and different doses of GPs and GP–Zn (II) complexes; (**B**) Migration of macrophages to the lateral line revealed by Image-Pro Plus software. Data are presented as mean ± standard deviation. Different letters (a, b, c and d) indicate significant differences between the number of fluorescent cells (*p* < 0.05).

Figure 5 showed the mitigation of CuSO_4_-induced inflammatory response in zebrafish by GPs and GP–Zn (II) derivatives. Compared with the normal group, CuSO_4_ treatment led to a 30.6-fold increase in the level of migrating and accumulating inflammatory cells in zebrafish larvae (*p* < 0.05). This indicated the successful establishment of a CuSO_4_-induced inflammation model in zebrafish [33]. However, the administration of ibuprofen, GPs and GP–Zn (II) complexes significantly alleviated this inflammatory damage (Figure S1). Ibuprofen exhibited the most effective inflammation-inhibiting effect, with a 60.13% decrease in the number of migrant inflammatory cells compared with CuSO_4_-induced inflammatory zebrafish (*p* < 0.05). In the concentration range of 5–100 µg/mL, the anti-inflammatory activity of GPs enhanced with increasing concentration (with a 22.88–45.10% decrease in the number of migrant inflammatory cells compared with zebrafish in the model group), while the opposite trend was observed for the GP–Zn (II) complexes (with a 43.14–58.17% reduction in the number of migrant inflammatory cells compared with the model group). At the same concentration, GP–Zn (II) complexes exerted significantly (5 μg/mL) or insignificantly (10 and 100 μg/mL) higher anti-inflammatory activity than GPs (*p* < 0.05). Therefore, the mitigation of GPs on CuSO_4_-induced inflammatory response in zebrafish was greatly enhanced by the chelation of Zn (II).

#### 3.9.2. Effect of GPs and Their Zn (II) Derivatives on the Expression Levels of Inflammation-Associated Cytokines

**Figure 6:**
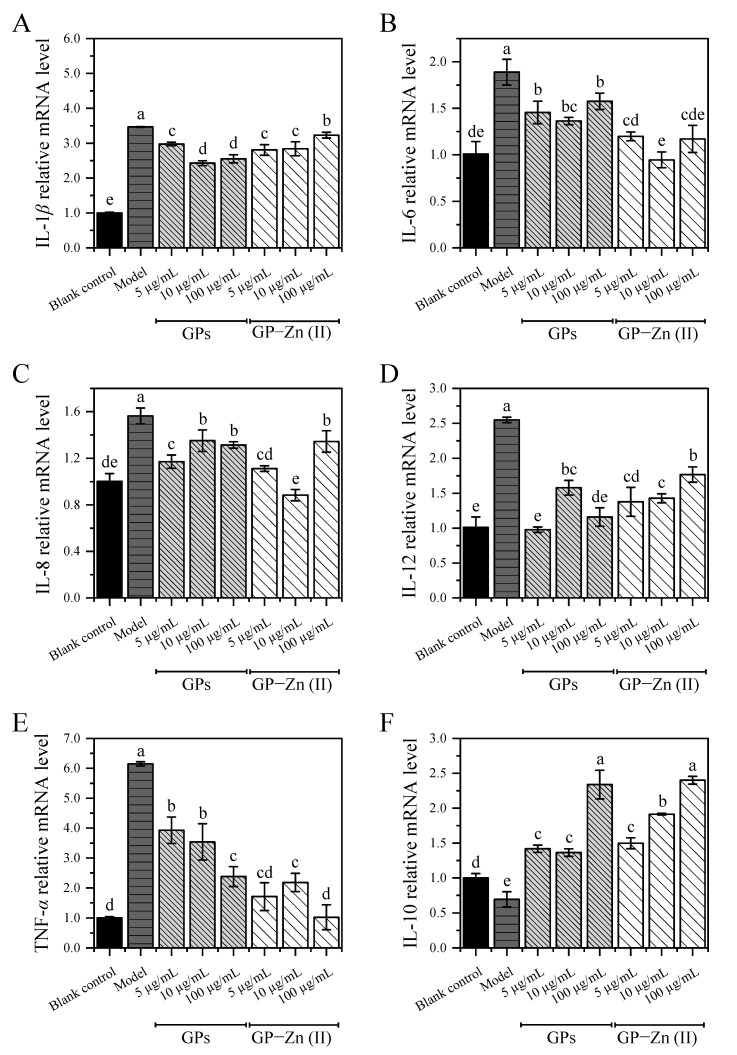
Effect of GPs and GP–Zn (II) complexes on the expression levels of inflammation-related cytokines in zebrafish. (**A**) IL-1*β*; (**B**) IL-6; (**C**) IL-8; (**D**) IL-12; (**E**) TNF-*α*; (**F**) IL-10. Data are presented as mean ± standard deviation. Different letters (a, b, c, d and e) indicate significant differences between the expression levels of inflammatory cytokines (*p* < 0.05).

The key role of the NF-*κ*B/MAPK signaling pathway in the pathogenesis and development of inflammation has been demonstrated in several studies [22,34,35,36]. The phosphorylation of some transcription factors and proteins, such as NF-*κ*b, I*κ*b-*α*, p38 and JNK, is the typical feature of the activated NF-*κ*B/MAPK signaling pathway [34]. As there are very limited reliable antibodies against zebrafish, the bioactive mechanisms of functional components in most studies based on zebrafish models are mainly evaluated by detecting the expression levels of inflammation-related cytokines using qRT-PCR (with high sensitivity, specificity and accuracy). Therefore, the expression levels of five pro-inflammatory cytokines (IL-1*β*, IL-6, IL-8, IL-12 and TNF-*α*) and one anti-inflammatory cytokine (IL-10) in the NF-*κ*B/MAPK signaling pathway in zebrafish were examined by qRT-PCR to explore the potential anti-inflammatory mechanisms of GPs and GP–Zn (II) complexes. As shown in Figure 6, the mRNA expression levels of IL-1*β*, IL-6, IL-8, IL-12 and TNF-*α* were upregulated significantly by 0.56–5.14-fold in CuSO_4_-induced zebrafish compared with the control group, accompanied by a significant decrease in the expression of IL-10 (*p* < 0.05). This suggested that the treatment of CuSO_4_ induced a severe inflammatory response by disrupting the balance of pro-inflammatory and anti-inflammatory cytokines in the NF-*κ*B/MAPK signaling pathway. After the administration of GPs and their Zn (II) derivatives (concentration: 5–100 µg/mL), the mRNA expression levels of five pro-inflammatory cytokines (IL-1*β*, IL-6, IL-8, IL-12 and TNF-*α*) were effectively inhibited by 6.88–29.91%, 16.59–49.94%, 13.63–43.45%, 30.61–61.64% and 36.09–83.37%, respectively, while the expression of anti-inflammatory cytokine (IL-10) was significantly promoted by 0.96–2.46-fold in zebrafish. It was reported that ginger polysaccharides alleviated the intestinal inflammation symptoms and inhibited the production of pro-inflammatory cytokines (e.g., TNF-*α*, IL-1*β* and IL-6) in dextran sulfate sodium-induced ulcerative colitis mouse model, which was in accordance with our findings [37]. Among the six pro-/anti-inflammatory cytokines, the changes of TNF-*α* stimulated by CuSO_4_ but alleviated by GPs and their Zn (II) derivatives were the most significant, with a 5.14-fold increase in the expression level compared with the control group, but a 36.09–83.37% down-regulation compared with CuSO_4_-induced inflammatory zebrafish. In the low and high dose groups of GP–Zn (II) complexes (5 µg/mL and 100 µg/mL), the expression levels of TNF-*α* were close to those of the blank control group, with no significant differences (*p* > 0.05).

Although GP–Zn (II) complexes exerted significantly (5 μg/mL) or insignificantly (10 and 100 μg/mL) higher anti-inflammatory activity than GPs, some inflammatory cytokines did not follow the similar trend. Overall, the modified GP–Zn (II) derivatives exhibited a higher regulation on the expression levels of IL-6, IL-8, TNF-*α* and IL-10, but a lower inhibition on the expression of IL-1*β* and IL-12. The reason might be that GPs and their Zn (II) complexes had different regulatory mechanisms on the inflammatory response or their main regulatory pathways were different, despite having some of the same targets [22]. Notably, the regulation of the mRNA expression levels of these inflammation-related cytokines by GPs and GP–Zn (II) complexes was not always dose-dependent, which was similar to other studies [22,38]. In general, the dose-bioactivity relationship showed a parabolic pattern, with an optimal dose at the apex. As the sample concentration increased from 5 μg/mL to 100 μg/mL, the continuously increasing inhibition on the pro-inflammatory cytokines suggested that the optimal dose was closer to 100 μg/mL (the apex of the parabola), while the trend of increasing and then decreasing modulation on inflammation indicated that the optimal dose was closer to 10 μg/mL. Therefore, the effect of GPs and GP–Zn (II) complexes on these inflammation-related cytokines was not always dose-dependent. In summary, GPs and GP–Zn (II) complexes might exert anti-inflammatory activity by down-regulating the mRNA expression levels of pro-inflammatory cytokines (IL-1*β*, IL-6, IL-8, IL-12 and TNF-*α*) and upregulating the expression of anti-inflammatory cytokine (IL-10).

## 4. Conclusions

In this study, GP–Zn (II) complexes were successfully synthesized by thermal reaction, with a Zn content of 21.17 ± 0.25 mg/g. The obtained complexes consisted dominantly of glucose and galactose in the mass proportion of 95.10:2.10, with a molecular weight of 4.90 × 10^5^ Da. Structural characterization suggested that the modification of GPs by Zn (II) occurred mainly on the hydroxyl groups, and this interaction reduced the crystallinity and decreased the asymmetry of GPs, with a slight effect on the thermal stability. In a CuSO_4_-induced inflammation model in zebrafish, the administration of GPs and their Zn (II) derivatives effectively alleviated the inflammatory response *via* down-regulating the mRNA expression levels of pro-inflammatory cytokines (IL-1*β*, IL-6, IL-8, IL-12 and TNF-*α*) and upregulating the expression of anti-inflammatory cytokine (IL-10). Furthermore, the introduction of Zn (II) enhanced the inflammation-inhibiting effect of polysaccharides. Therefore, GP–Zn (II) complexes could be applied as a candidate anti-inflammatory agent for the treatment of chronic inflammation-related diseases.

## Data Availability

Not applicable.

## Supplementary Materials

The following supporting information can be downloaded at: https://www.mdpi.com/article/10.3390/antiox11122331/s1, Table S1: Primers used for qRT-PCR analysis. Figure S1: Representative images of zebrafish treated with CuSO_4_, ibuprofen and different doses of GPs and GP–Zn (II) complexes.
