# Supplementary material for: Preparation and Characterization of Ginger Peel Polysaccharide–Zn (II) Complexes and Evaluation of Anti-Inflammatory Activity
Source: Antioxidants (Basel). 2022 Nov 25;11(12):2331. doi: 10.3390/antiox11122331 (PMC9774354; doi:10.3390/antiox11122331)
Supplement: Supplementary file 1 [file antioxidants-11-02331-s001.zip › antioxidants-2033288-supplementary.pdf]

Supplementary Data

Preparation and Characterization of Ginger Peel Polysaccharide–Zn (II) Complexes and Evaluation of Anti-Inflammatory Activity

Wenwen Li [1], Zhichang Qiu [1], Yue Ma [2], Bin Zhang [1], Lingyu Li [1], Qiulin Li [3], Qiuxia He [4,*], Zhenjia Zheng [1,*]

[1] Key Laboratory of Food Processing Technology and Quality Control of Shandong Higher Education Institutes, College of Food Science and Engineering, Shandong Agricultural University, Tai'an 271018, China.
[2] Institute of Agri-food Processing and Nutrition, Beijing Academy of Agriculture and Forestry Sciences, Beijing Key Laboratory of Agricultural Products of Fruits and Vegetables Preservation and Processing, Key Laboratory of Vegetable Postharvest Processing, Ministry of Agriculture and Rural Affairs, Beijing 100097, China.
[3] School of Materials Science and Engineering, Suzhou University of Science and Technology, Suzhou 215011, China.
[4] Science and Technology Service Platform of Shandong Academy of Sciences, Qilu University of Technology (Shandong Academy of Sciences), Jinan 250103, China.

* Corresponding authors: heqx@sdas.org (Q.H.); zhengzhenjia@sdau.edu.cn (Z.Z.)

1. Material and Methods

1.1 Effect of GPs and GP–Zn (II) Complexes on the Expression Levels of Inflammation-Related Cytokines in Zebrafish

Table S1 Primers used for qRT-PCR analysis.

Gene	Upstream primer	Downstream primer
IL-1*β*	5‘-CTCAGCCTGTGTGTTTGGGA-3’	5‘-GGGACATTTGACGGACTCG-3’
IL-6	5‘-ACGACATCAAACACAGCACC-3’	5‘-TCGATCATCACGCTGGAGAA-3’
IL-8	5‘-CAAGAACCATTGGGATGAAGGAC-3’	5‘-CCTTCAGTAGCCTCTGTCCTTGT-3’
IL-12	5‘-ACTCCTACAAGCCCAGCAC-3’	5‘-GGACACTCGGTCGTCAAAC-3’
TNF-*α*	5‘-GCTGGATCTTCAAAGTCGGGTGTA-3’	5‘-TGTGAGTCTCAGCACACTTCCATC-3’
IL-10	5‘-GGAGACCATTCTGCCAACA-3’	5‘-CATTTCACCATATCCCGCT-3’
β-actin	5′-CTCCGGTATGTGCAAAGC-3′	5′-CCATCACTCCCTGATGTCT-3′

2. Results and Discussion

2.1 Evaluation of the Anti-Inflammatory Activity of GPs and GP–Zn (II) Complexes and Underlying Mechanisms

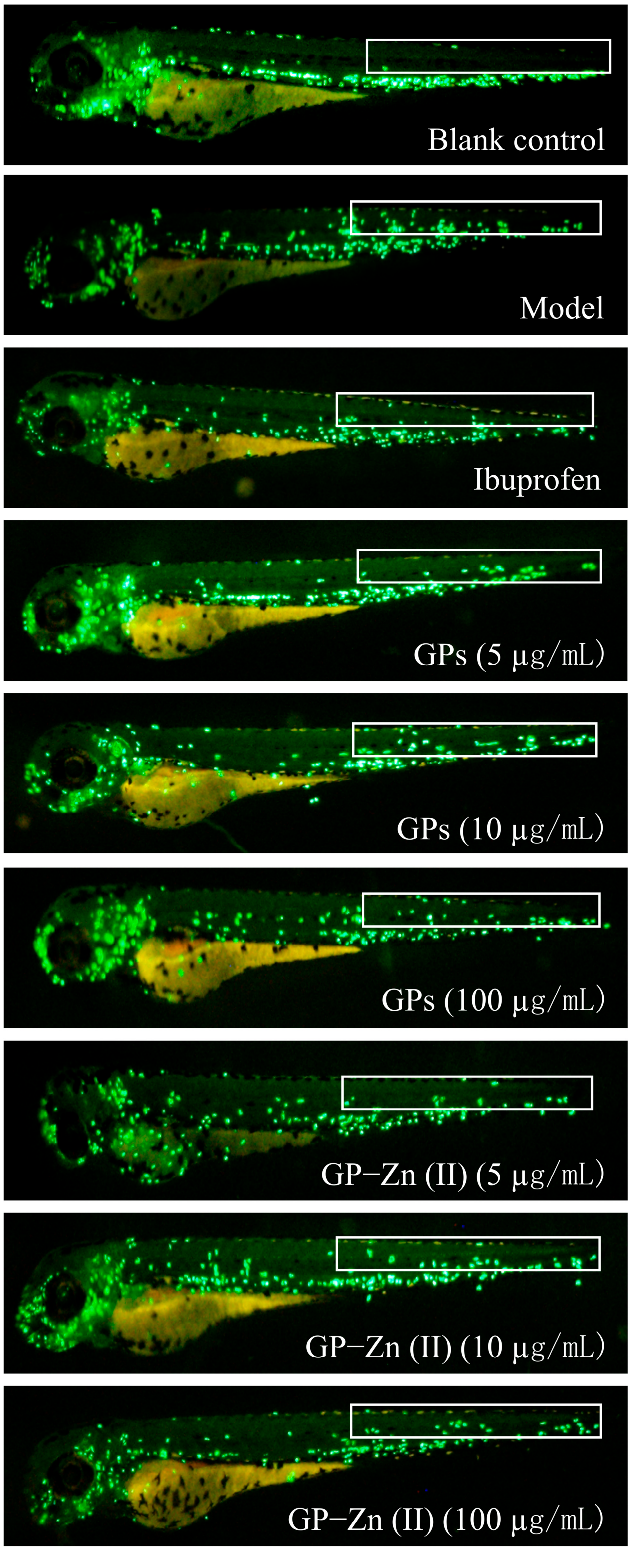

Figure S1. Representative images of zebrafish treated with $CuSO_4$, ibuprofen and different doses of GPs and GP–Zn (II) complexes.
